# Women's awareness of ovarian cancer risk factors and symptoms in Western Iran in 2020–2021

**DOI:** 10.1186/s12905-022-01779-x

**Published:** 2022-05-25

**Authors:** Babaei Maryam, Salmani Fatemeh, Kariman Nourossadat, Nasiri Saeideh, Ozgoli Giti

**Affiliations:** 1 Student Research Committee, Department of Midwifery and Reproductive Health, School of Nursing and Midwifery, Shahid Beheshti University of Medical Sciences, Tehran, Iran; 2 Department of Epidemiology and Biostatistics, Social Determinants of Health Research Center, Faculty of Health, Birjand University of Medical Sciences, Birjand, Iran; 3 Midwifery and Reproductive Health Research Center, Department of Midwifery and Reproductive Health, School of Nursing and Midwifery, Shahid Beheshti University of Medical Sciences, Tehran, Iran; 4 Department of Midwifery, School of Nursing and Midwifery, Kashan University of Medical Sciences, Kashan, Iran

**Keywords:** Awareness, Ovarian neoplasms, Risk-factors, Women, Symptoms

## Abstract

**Objectives:**

This study aimed to investigate awareness of women living in the western region of Iran about warning signs and risk factors for ovarian cancer.

**Methods:**

This cross-sectional, descriptive-analytical study was conducted in Sanandaj and Kermanshah, Iran, 2020 and 2021. In this study, 1081 women aged 18–70 years were selected as the sample to fill out the electronic version of the Ovarian Cancer Awareness Measure (OCAM) to identify the significant variables of ovarian cancer warning signs and risk factors. The obtained data were statistically analyzed by descriptive statistics and ordinal logistic regression in SPSS 19.

**Results:**

In this study, 60.9% of the participants had medium awareness of the subject. The results indicated that participants with higher educational attainment (P < 0.05) and those with a history of cancer (P < 0.001) showed higher knowledge of cancer. The lowest level of awareness of symptoms was associated with acute symptoms such as dysphagia (swallowing problems) most days, persistent bloating, a sense of abdominal fullness or heaviness, and frequent urination. Moreover, the lowest awareness of risk factors was related to the history of IVF treatments and the application of talcum powder to the genital area.

**Conclusion:**

The study findings showed that women living in the western region of Iran have moderate awareness of ovarian cancer and insufficient knowledge of cancer warning signs; this suggests that it is necessary to train Iranian women to raise awareness of the signs and risk factors for ovarian cancer.

## Introduction

Ovarian cancer is the third most common cancer and the third leading cause of cancer death among women in Low/Medium HDI Countries; it is considered one of the deadliest cancers. This disease affected 313,959 women and took the lives of 207,252 women worldwide in 2020. The most recent estimates reveal that the number of new cases of ovarian cancer per year will reach 371,000 by 2035; in addition, the number of women diagnosed with this disease will increase by 47% to 434,184 associated deaths reach 293,039 by 2040 [1]. Ovarian cancer is the most common gynecological cancer affecting women in Iran. In 2020, the number of deaths related to ovarian cancer in Iran was 1269, which was high compared with the 1966 newly diagnosed cases [2].

A variety of factors cause ovarian cancer, including lifestyle, environmental and genetic factors [3] Sharifian et al. reported that ovarian cancer's prevalence and mortality rate had an ascending trend in Iran from 1999 to 2013 [4]. Although the prevalence of ovarian cancer is still low in Iran, the rising trend of obesity, physical inactivity, unhealthy diet, older age of menopause, decreasing parity, and lactation are some factors that may increase cancer's prevalence in the future [5]. Although the incidence of ovarian cancer appears to be lower than that of breast cancer, it is three times more lethal [6]. Most women with ovarian cancer (approximately 75%) are diagnosed when cancer progresses to a more complicated treatment stage. Since cancer prognosis and survival rates differ depending on the stage of the disease when diagnosed, the prognosis and survival rates improve if the disease is diagnosed as early as possible [7].

Currently, the best biological diagnostic tool seems to be a combination of CA125 and HE4 levels to predict the risk of ovarian cancer in patients suspected of having benign ovarian tumors [8]. However, there is no Diagnostic method that meets the requirements for the early detection of ovarian cancer in the general population [9].

Contrary to popular belief, there is ample evidence that ovarian cancer has warning signs and symptoms, referred to as a "key message." Raising public awareness of ovarian cancer can be helpful in early diagnosis, and adequate knowledge of the disease's risk factors can help women recognize symptoms and encourage them to seek and adhere to medical treatments [10]. One of the primary reasons for the late diagnosis of cancer is the lack of general knowledge about cancer's early signs and symptoms [11].

Increasing women's awareness of cancer symptoms and risk factors while the incidence of its growth can effectively prevent that progression and reduce the cost of treatment imposed on the healthcare system [12]. Raising awareness and attitude, social norms, or self-efficacy can significantly alter behavior to improve lifestyle, modify ovarian cancer risk factors, and reduce the potential gap between being a sign and diagnosing ovarian cancer in women [13].

According to a literature review, no study has been conducted in Iran to assess the level of knowledge about ovarian cancer. Most studies on ovarian cancer in Iran have focused on the prevalence and risk factors.

## Methods

This cross-sectional, descriptive-analytical study was conducted on women in Kermanshah and Kurdistan provinces, Iran, from September 2020 to June 2021. The participants of this study were selected based on the multi-stage and network sampling methods. First, a list of cities and towns in Kermanshah and Kurdistan provinces was prepared. Then a quota was allocated to each province according to the number of cities, so three cities from Kurdistan Province and four from Kermanshah Province with a higher population were selected. Finally, Sanandaj, Marivan, and Saqez from Kurdistan Province and Kermanshah, Islamabad Gharb, Sarpol Zahab, and Javanroud from Kermanshah Province were selected for sampling. Midwives working in urban health centers in each city who were willing to participate in this study were briefed on the questionnaire and how it should be completed. After the online multi-stage sampling, the designed questionnaire's link was provided to midwives online via WhatsApp. After briefing them on the research objectives and procedures, the selected midwives then provided the link to the patients who were eligible to participate in the study. In cases where the participants could not complete the online form, the midwife asked the participant questions, and the answers were entered into the online form. Sampling was done at this stage by Convenience Sampling Method.

The inclusion criteria were 18–70 years, no history of ovarian cancer, and willingness to participate in the study. The sampling process continued until the intended sample size was completed. Participants were asked to complete the questionnaire after expressing informed consent. The questionnaire was created in Google Forms, and the link was sent to participants via WhatsApp. The first section of the online questionnaire thoroughly briefed participants on the objectives and how to complete them. The participants were also assured that their information would be kept confidential. The sample size was calculated using the formula n = (Z_1−α/2_ + Z_β_)^2^ p (1 − p)/d^2^ assuming a type-1 error of 0.05, power of 80%, and a proportion of women with the excellent knowledge of ovarian cancer (p) of 25% [14]. The calculated sample size was 918 (d: 0.04); considering that about 30% of questionnaires would be incomplete, the sample size was 1150. Cancer Awareness Measure (CAM) is a scale developed in the UK to help measure cancer awareness, identify risk factors related to poor awareness, and develop and evaluate interventions to promote cancer awareness. There are different versions of this valid questionnaire for different cancers applicable to face-to-face, online, or telephone interviews and self-administration [10, 15]. This questionnaire consisted of 35 items in "warning signs" (10 multiple-choice items and one open-ended item), "delay in seeking medical help" (1 open-ended item), "ovarian cancer age" (one multiple-choice item), "risk factors" (12 multiple-choice items and one open-ended item), "NHS screening programs" (8 items), and "confidence in diagnosing the symptoms of ovarian cancer" (one multiple-choice item).

The questionnaire was independently translated from English to Persian (translation forward) by two experts in the first step, after obtaining permission from the Ovarian Cancer Awareness Measure (OCAM). The research team then combined the two translation versions to create a single copy. Two specialists separately translated the final form into English (translation backward), and the research team merged the two English translations into a single copy [16]. The tool was then reviewed by 10 experts (all of whom had a Ph.D. in Reproductive Health and Health Education and were knowledgeable and experienced in the measurement of instruments and ovarian cancer) to provide their corrective feedback on the grammar and vocabulary quality of the text, item arrangement, and scoring system. This questionnaire version was tested in a pilot study on ten women who qualified for the study, and the last changes were made based on participant feedback. The questionnaire items were reviewed and modified for cultural adaptation to eliminate any wrong items for Iranian culture. Open-ended questions were eliminated from the questionnaire due to the lack of appropriate facilities for face-to-face interviews. The open-ended item about health-seeking practices was converted into a multiple-choice item to facilitate responses. The answer options included instantly, one week, two weeks, as soon as possible, one month, a few months later, and never. In this study, a modified version of the OCAM was used. The questionnaire included two sections.; the first section consisted of items about demographic information such as language, age, marital status, employment status, educational attainment, place of residence, and having a relative, friend, or family member with cancer (considering the cancer type). The second part contained items measuring "warning signs" (10 multiple-choice items), "delay in seeking medical help" (one multiple-choice item), "ovarian cancer age" (one multiple-choice item), "risk factors" (12 multiple-choice items), and "confidence in diagnosing the symptoms of ovarian cancer" (one multiple-choice item) as well as four items concerning national awareness screening programs. Each correct response was assigned a score of 2 to determine awareness about the symptoms, risk factors, typical ages of developing ovarian cancer, and national screening programs. A score below 16, between 16 and 36, and over 36 was regarded as low, moderate, and suitable knowledge of ovarian cancer, respectively. The division is expressed as poor awareness for people in the lower 33% of the population score, between 33 to 66% of the moderate, and above 66% of the good score in the study population.

Ten experts (reproductive health and health education specialist) were asked to evaluate the validity of the questionnaire's qualitative and quantitative content. The questionnaire was then modified based on their comments on the grammar, vocabulary, necessity, syntax, collocation, and scoring system. The Likert scale was employed to assess relevance, simplicity, clarity, and necessity for each item, and the content validity ratio (CVR) was calculated quantitatively. The mean CVR was equal to 0.88, whereas the content validity index (CVI) was more significant than 0.79. Internal correlation and test–retest reliability were used to assess the stability of the questionnaire. The most commonly used method for determining internal correlation is Cronbach's alpha, which ranges from 0 to 1. It is appropriate to have an internal correlation more significant than 0.7 [17]. Cronbach's alpha for the entire questionnaire was 0.88, indicating the appropriate reliability of this tool for the Iranian population. In the retest technique, 20 qualified participants completed the questionnaire twice at a 2-week interval. The intraclass correlation coefficient (ICC) was used to assess the instrument's test–retest reliability, which was calculated to be 0.86. An ICC of 0.8 or higher indicates excellent stability [18]. The quantitative variables in this study were defined by mean, standard deviation, and interquartile range, whereas the qualitative variables were presented by frequency and percentage. The percentage of awareness was shown using bar charts, and ordinal logistic regression was used to determine the predictors of awareness. The data were statistically analyzed in SPSS-19 at the 0.05 level of significance. The rank logistic regression was used in this study to measure the response variable, i.e., knowledge (good, moderate, and low). To predict the higher chances of knowing age, educational attainment, history of cancer (the genital tract, breast, and intestine in family, friends, and close relatives(, dialect, and marital status were added separately and then concurrently to the model (ordinal logistic regression).

## Findings

A total of 1300 women volunteered for this study, but only 1081 completed the questionnaire.

The sample size obtained is more than the sample size required for the study, and Incomplete questionnaires did not affect the study results.

**Fig. 1:**
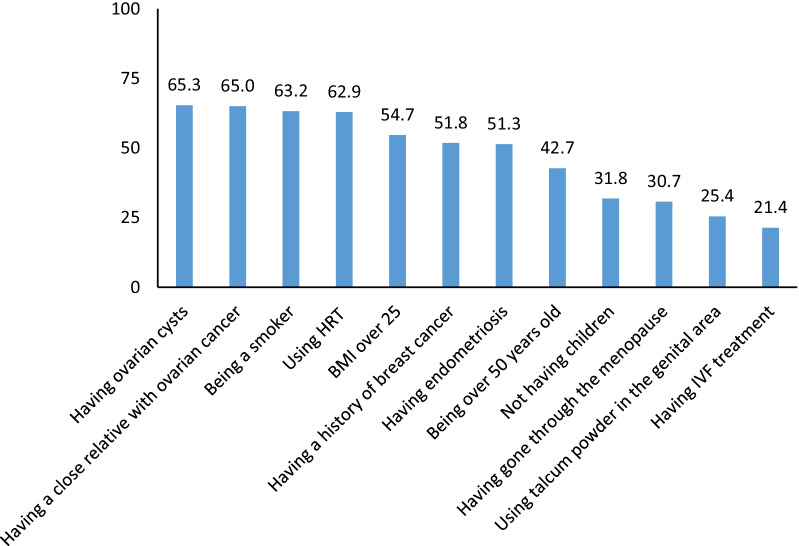
Distribution of participants responses to the risk factors (n: 1081)

**Fig. 2:**
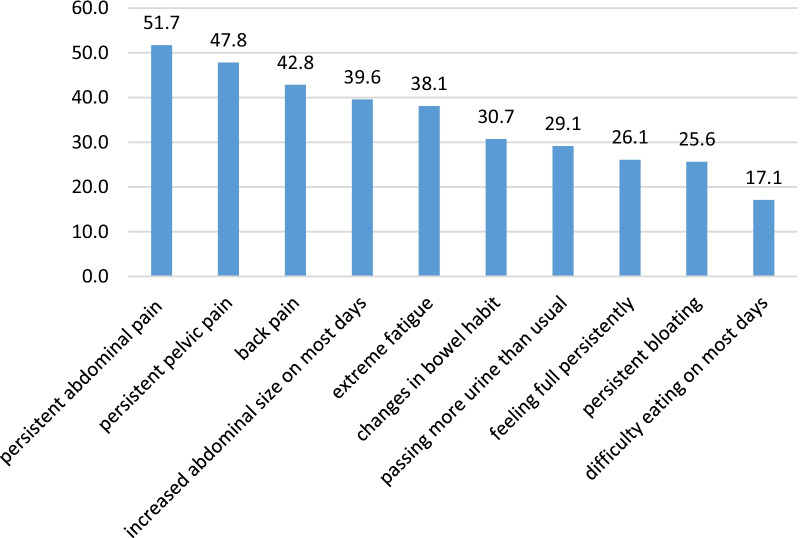
Distribution of participants responses to the warning signs (n: 1081)

**Fig. 3:**
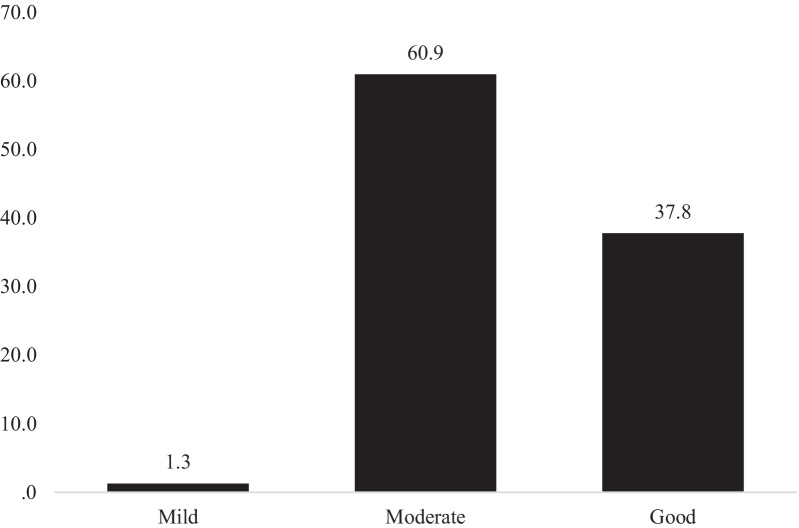
Percent of the level of knowledge on ovarian cancer

Six hundred five samples were from different cities in Kurdistan Province, with Sanandaj having the highest number of participants. Moreover, the highest number of participants in Kermanshah Province were from the city of Kermanshah. The mean age of participants was 32.63 ± 8.13 years. The majority of the participants (n: 772, 71.3%) were Kurdish speakers. 58.3%( n: 631) were married, and most of them (n: 519, 47.9%) had a bachelor's degree. The data showed that most participants (n: 664, 61.3%) had no familial history of cancer (Table 1). The frequency and percentage of ovarian cancer risk factors and warning signs are presented in Figs. 1 and 2. When the self-confidence of participants was tested, the results indicated that 49 participants (4.5%) were utterly sure, 431 (39.8%) were not sure, 363 (33.5%) were not at all sure, and 240 (22.2%) were found to be less confident in identifying ovarian cancer. The results also showed that 470 participants (43.4%) visited medical centers immediately after diagnosis, 96 (8.9%) at the first opportunity, 152 (14%) within one week, 29 (2.7%) within two weeks, 136 (12.6%) within one month, 83 (7.7%) with a delay of several months, and 117 (10.8%) never. The mean and standard deviation of awareness scores were 10.57 ± 4.73, 0.87 ± 0.84, 16.1 ± 4.25, and 6.74 ± 1.44 for warning symptoms, age, risk factors, and screening dimensions, respectively. The overall awareness score was obtained at 34.29 ± 7.63 (Table 2). In this study, 60.9% and 37.8% of the participants had a moderate and good awareness of ovarian cancer (Fig. 3). The results indicate that women with higher educational attainment and a history of cancer were more likely to be more aware of ovarian cancer (p < 0.05). The multiple-order logistic model demonstrated that educational attainment (a high school diploma: OR = 0.39, P-value < 0.001 and a bachelor's degree: OR = 0.68, P-value = 0.01) and history of cancer (OR = 0.62, P-value < 0.001) were other factors affecting participants' awareness. In this regard, participants with a high school diploma were 0.39 times less likely to be aware of cancer than those with a master's degree or a Ph.D. In addition, participants with a bachelor's degree were 0.68 times less likely to have a high level of cancer awareness than those with a master's or a Ph.D. The results showed that participants (without a history of cancer) were less likely (0.62%) to have a high level of cancer awareness than those with a history of cancer.

**Table 1:** Demographic characteristic of participants (n: 1081)

Variables	Mean	SD
Age	32.63	8.13

	n	%
*Dialect*		
Kord	772	71.3
Fars	278	25.7
Else	33	3.0
*Marital status*		
Married	631	58.3
Single	407	37.6
Widowed and divorced	45	4.2
*Education level*		
Illiterate and undergraduate	63	5.8
High school diploma	195	18.0
Bachelor degree	519	47.9
Masters and PhD	306	28.3
*History of cancer*		
Negative	664	61.3
Positive	419	38.7

**Table 2:** Mean, SD, IQR, and confidence interval of knowledge on ovarian cancer

Variables	Mean	SD	Confidence interval of the mean		IQR*
			25%	975%	
Warning signs dimension	10.57	4.73	10.29	10.85	6
Ovarian cancer age dimension	0.87	0.84	0.82	0.92	2
Risk factors dimension	16.10	4.25	15.85	16.35	6
National screening programs dimension	6.74	1.44	6.66	6.83	9
Total score	34.29	7.63	33.83	34.75	9

*Interquartile range

## Discussion

More than 50% of participants had moderate awareness; meanwhile, participants with higher educational attainment and those with a family history of cancer exhibited higher awareness of cancer. Persistent abdomen pain and persistent pelvic pains were the most recognized symptoms and signs by women among the warnings. On the other hand, the lowest level of awareness was related to "dysphagia (swallowing problems) most days," "persistent bloating," "a sense of abdominal fullness or heaviness," and "frequent urination."

In a study by Low et al. in the UK, the lowest level of awareness of warning signs was related to dysphagia (swallowing problems) most days, a persistent sense of fullness, and frequent urination [19]. Elshami et al. reported that the least recognized signs and symptoms of ovarian cancer among Palestinian women were a persistent sense of fullness and dysphagia (swallowing problems) most days. They hence concluded that Palestinian women had a low level of awareness about ovarian cancer symptoms [20]. Approximately 25% of ovarian cancer patients experience gastrointestinal problems within a year of being diagnosed. Ovarian cancer should be considered in the differential diagnosis of women with goiter symptoms, as it may be effective in the treatment process [21]. Goff et al. (2004) defined three symptoms (increased abdominal size or bloating, pelvic or abdominal pain, and dysphagia or a sense of quick fullness) for ovarian cancer [22, 23]. The study findings demonstrated that the minimum level of awareness was related to the key symptoms of ovarian cancer. Only 51.7% of participants knew that persistent pelvic and abdominal pain is among the symptoms of ovarian cancer, which can be a severe concern. Some symptoms of ovarian cancer, such as abdominal and pelvic pain, constipation, and increased abdominal size, may be more severe and prevalent among women with ovarian cancer than in the general population [23].

Additionally, some symptoms of ovarian cancer may be confused with those of benign and more prevalent diseases, which can prevent timely visits for medical treatments [24]. Early visits to medical centers after observing any symptoms of ovarian cancer can be associated with better therapeutic outcomes [25]. In this study, 7.7% of participants stated that they would wait several months before visiting a medical center if they suspected the warning signs of ovarian cancer, and 10.8% of them said that they would never seek medical care.

There are several reasons for not seeing or delaying seeing a doctor due to the cultural context of the western region of Iran, such as fear of cancer, shame, embarrassment, lack of health insurance and financial support, not seeing oneself at risk, belief in fate, lack of time due to family and career commitments, access to healthcare facilities, quality of health services, and forgetting [26, 27].

Since the 5-year prognosis of this disease with early diagnosis in stages 1 and 2 is more than 70%, and whenever the diagnosis is delayed, survival rates are reduced to 20 to 40%. Therefore, early referral due to increasing awareness about warning signs and danger and how to treat the disease can effectively affect patient survival [8]. The results also showed that 73.3% of participants were not sure about the symptoms of ovarian cancer. The study findings suggested a need for further in-depth investigations into why women do not visit or visit late medical centers after observing a symptom of ovarian cancer. Such studies are critical because the warning signs of ovarian cancer can be confusing for women. Because symptoms such as abdominal pain, anorexia, and a constant sense of fullness have been associated with the increased mortality risk [25], it is critical to raise women's awareness of the warning signs of ovarian cancer. The lowest level of awareness about risk factors was related to IVF treatment history and the application of talcum powder to the genital area. By contrast, the highest level of awareness about risk factors was related to a history of ovarian cysts and a family history of ovarian cancer. The participants were more aware of risk factors than the warning signs of ovarian cancer. Since there is no effective prevention strategy for ovarian cancer, and genetic and environmental factors influence the prevalence of this disease, training can help reduce the prevalence and mortality rate of ovarian cancer [24], and the raising awareness gained from training can enable women to improve their health control [25]. Since the study results demonstrated a link between educational attainment and awareness about ovarian cancer, women with higher educational attainment were more aware of ovarian cancer. Educational attainment is one of the most important determinants of cancer awareness [28]. Higher educational attainment allows women to become more aware of their environment and health and increases their chances of accessing health information that will help them follow healthier lifestyles [29]. Some studies conducted in low-income countries showed a significant relationship between the educational attainment of women and their awareness of ovarian cancer, as women with higher educational attainment were more aware of ovarian cancer. This result is consistent with the findings of this study [30, 31]. In this study, women with a history of Female cancers were more aware of ovarian cancer. The emotional experience of cancer among one's relatives can significantly affect one's level of anxiety and sensitivity to the warning signs and risk factors for cancer [32]. Individuals who understand the risk of cancers are more likely to decide to seek screening programs, behavioral changes, or medical treatment [33]. A family history of cancer is one of the most common reasons some people understand higher cancer risk.

The main strength of this study is that it was one of the first studies to deal with women's awareness of the risk factors and warning signs of ovarian cancer and their knowledge of screening methods. However, this study had some limitations in terms of design and procedure. For example, the data were collected via an online questionnaire due to the COVID-19 pandemic. Although online data collection tools bring advantages such as lower cost [34], higher quality of gathered data [35], and shorter data entry [36], one of the limitations of such tools is the possible limited internet access for some community members. However, on the other hand, the use of the internet caused access to the young population, which is not usually easy to access. Since only 52% of people are internet users with moderate skills in the studied regions, Kurdistan and Kermanshah provinces of Iran [37], it was not possible to measure the awareness of women who had no internet access or knowledge. Moreover, because only 258 members of the study population (23.8%) had a high school diploma or a higher degree, future studies are recommended to evaluate awareness about ovarian cancer among women with lower educational attainment.

## Conclusion

The study findings suggested that women living in the western region of Iran have moderate awareness of ovarian cancer and insufficient knowledge of its warning signs. The results showed that the lowest knowledge of warning signs was related to dysphagia (swallowing problems) most days, persistent bloating, constant sense of abdominal fullness and heaviness, and frequent urination. Furthermore, the lowest knowledge of risk factors was related to the application of talcum powder to the genital area and history of IVF treatment. Given that the awareness of warning signs, risk factors, and age of cancer incidence can facilitate the early diagnosis of ovarian cancer, training women in these issues is necessary to raise awareness. The results of this study can also effectively evaluate and design prevention programs for ovarian cancer.

## Data Availability

The datasets generated and/or analysed during the current study are not publicly available due they contain information that could compromise research participant privacy but are available from the corresponding author on reasonable request.

## Abbreviations

OCAM: Ovarian Cancer Awareness Measure
CAM: Cancer Awareness Measure
